# Appealing dish names to nudge diners to more sustainable food choices: a quasi-experimental study

**DOI:** 10.1186/s12889-022-14683-8

**Published:** 2022-11-30

**Authors:** Anna Gavrieli, Sophie Attwood, Jonathan Wise, Eleanor Putnam-Farr, Paul Stillman, Scott Giambastiani, Jane Upritchard, Chavanne Hanson, Michiel Bakker

**Affiliations:** 1 Compass Group, North America, 2400 Yorkmont Road, Charlotte, NC 28217 USA; 2 Better Buying Lab, World Resources Institute, Thomas House, 84 Eccleston Square, London, SW1V 1PX UK; 3 Jones Graduate School of Business, Rice University, 1900 Rice Boulevard, Houston, TX 77005 USA; 4 Yale Center for Customer Insights, Yale School of Management, Yale University, Evans Hall, 165 Whitney Avenue, New Haven, CT 06511 USA; 5 Google LLC, 1600 Amphitheatre Pkwy, Mountain View, CA 94043 USA

**Keywords:** Plant-rich diets, Sustainable food choices, Dish titles, Language, Food service, Climate change

## Abstract

**Background:**

Promoting plant-rich diets, i.e., diets with significantly reduced amounts of animal products, including vegan and vegetarian, is a promising strategy to help address the dual environmental and health crises that we currently face. Appealing dish names could boost interest in plant-rich dishes by attracting diners’ attention to them. In this study, a systematic approach to naming plant-rich dishes with appealing descriptors was tested with a quasi-experimental design in four workplace, self-service, buffet-style cafeterias in Chicago, Sydney, São Paulo and Singapore.

**Methods:**

Three different plant-rich dishes were tested at each site. Appealing names were generated systematically through a workshop and emphasized the dish ingredients, origin, flavor and/or the eating experience. Each test dish appeared once in a four-week menu cycle where menu options changed on a daily basis. The cycle was then repeated four times (six times in Chicago) with the total number of showings for each dish to be four (six in Chicago). The dish names alternated between basic and appealing across dish repetitions. For each dish, the food taken per plate was estimated by weighing the overall food taken and dividing it by the plate count in the cafeteria. Data was analysed as percentage change from baseline (i.e., the first showing of each dish that always had a basic name) with linear mixed effects analysis using the lme4 package in R.

**Results:**

Overall, appealing dish names significantly increased the amount of food taken per plate by 43.9% relative to baseline compared to basic dish names (54.5% vs. 10.6% increase for appealing vs. basic names, respectively, *p* = .002). This increase corresponded to a 7% increase in actual grams of food taken per plate. Secondary analysis showed that the effect was site-specific to English-speaking countries only and that there was no substitution effect between plant-rich and meat dishes.

**Conclusions:**

The study tested an approach to creating appealing dish names in a systematic way and indicates that, in some settings, appealing dish titles are a relatively easy, scalable, cost-effective strategy that the food services sector can adopt to shift food choices towards more plant-rich, sustainable ones.

## Background

The dual threats of climate change and the rising prevalence of non-communicable diseases stand to diminish the quality of life and well-being of future generations [1–3]. Tackling these issues requires immediate action on a global scale. A critical component of reducing both climate change and non-communicable diseases is shifting our food system to be more plant-rich (i.e., towards diets with significantly reduced amounts of animal products, including vegan and vegetarian) and environmentally sustainable [4, 5]. In this context, the general population of countries or cultures that follow red meat-heavy diets need to shift to more plant-rich choices, as red meat production is a primary contributor to greenhouse gas emissions and diets high in processed meat are often related to adverse health outcomes [6, 7]. However, many of the proposed solutions to encourage changes in dietary choices are costly or politically difficult to implement (e.g., taxes on certain foods) or rely on effortful self-control on behalf of consumers which can be challenging to maintain [8, 9].

Appealing language, that is, the use of descriptors that evoke positive attitudes and grab attention, is a marketing tool proven to influence consumers’ choices. Research shows that words and language can influence many priority behaviors in the field of public health: from vaccination to smoking cessation [10, 11]. Notably, however, plant-rich, healthy dishes are often described in restaurants or social media using less appealing language than their meat-rich counterparts [12, 13]. This may lead them to be perceived as less flavorful or indulgent, thereby deterring consumers from selecting these options [12–14].

Past work has provided initial evidence that renaming plant-rich menu items with more appealing descriptors emphasizing tasty and enjoyable attributes can increase selection of these dishes [15]. In one study, for instance, researchers in the US found that, whilst keeping the same recipe, renaming “carrots” to “twisted citrus-glazed carrots” led to a 25% increase in people selecting carrots [16]. Most of the research on this topic to date has been conducted in the US and the UK. It is unclear, though, if this approach is effective in other global locations. Anecdotal evidence from the World Resources Institute partners shows that appealing names work differently in Asian countries due to large differences in diet structure and norms around naming of food and this needs to be further confirmed. In addition, impeding the scalability of this strategy is the lack of a validated, systematic approach for food service operators to use to transform plant-rich names to more appealing ones.

In the present study, we developed a turnkey strategy that food service operators could use to create appealing dish names in a systematic manner, and assessed its efficacy in shifting consumers’ food choices in a field setting across different global locations and cultures. We hypothesized that renaming plant-rich dishes with more appealing descriptors generated through this systematic approach would a) primarily increase the amount of plant-rich food taken compared to basic names and b) secondarily reduce the amount of meat taken. In addition, we hypothesized that the effect would be c) location-specific and less pronounced in the Asian site as well as d) dish-specific and more pronounced for soups that were offered in opaque containers and they had their visual and olfactory cues removed.

## Methods

### Naming structure development

**Fig. 1:**
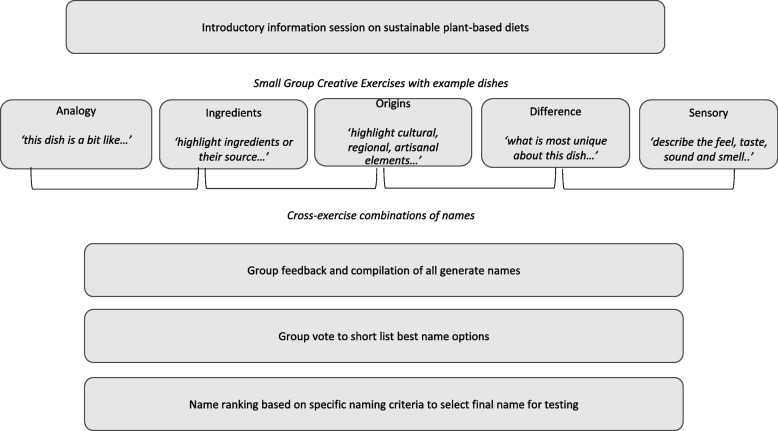
Schematic Representation of the Workshop

Before the intervention, we created a systematic naming approach to be delivered through a workshop based on past work published by the World Resources Institute [17]. Specifically, this work has shown that attributes which highlight provenance, spotlight flavor and emphasize a food’s look and feel are the most effective attributes to make the dish name more interesting and increase the likelihood for someone to select it [17]. The workshop consisted of five exercises that emphasized the dish’s analogy to other popular dishes; the dish ingredients or source of ingredients; the cultural and regional origin or preparation method of the dish; the difference from other dishes by emphasizing the dish’s most exciting characteristic; and its sensory appeal/eating experience (Fig. 1).

Then the research team conducted the workshop at each one of the four study sites (Chicago, Sydney, São Paulo, Singapore) with the food service operators. This workshop generated the appealing and basic names for the test dishes. Appealing names were created only for plant-rich dishes (not for the control dishes or the meat dishes that had only basic names - descriptions for the control and meat dishes to be found at the “Timeframe and test dishes” section below -). Before the workshop, Chefs responsible for creating the cafeteria menus, filled out a form with the test dishes details. These were made available to the workshop attendees during the workshop. The details provided for each test dish included the original dish title, a photo of the dish, the ingredients of the dish, the cooking instructions and the story of the dish, if any. For each workshop exercise, the food service attendees were asked to generate for each dish as many words/phrases as possible within 3 minutes using a post-it note per word/phrase. At the end, all post-it notes for each dish were placed on an empty wall and the attendees had to choose which words/phrases best describes the dish and compile three different names. In that way, three appealing names were generated for each plant-rich dish. These names were then ranked by the research team based on criteria on how well they reflected the workshop exercises and goals: a) how well someone could understand the generated name; b) if the dish name sounded to include lots of different components; c) if it sounded like it is good for health, of high quality; d) if someone could tell the regional or cultural origin; and e) if it was associated with a particular occasion. The name with the greatest score for each dish was then selected as the final test name (Table 1). Basic names were created in the same way with the appealing names asking attendees to generate as many basic names as they could for each test dish (plant-rich and meat-rich) within 3 minutes using post-its. At the end, the workshop attendees placed all post-its for each name on the wall and chose the name that they believed best described the dish.

**Table 1:** Plant-rich test dishes along with their basic and appealing names

Dish Type	Basic Name	Appealing Name
**Chicago**		
Main	Eggplant and Chickpea Stew	Nonna’s Garden Ragout
Main	Seitan Stew	Wine Simmered French Vegetable Medley
Soup	Tomato Soup	Provencal Slow-Roasted Herbal Tomato Soup
**Singapore**		
Main	Steamed Lentil & Couscous with Cauliflower	Soft Baked Cauliflower Tossed with Moroccan Grains
Side	Steamed Mixed Vegetables	Tricolore Summer Vegetables
Soup	Cauliflower Soup	French Smoked Cauliflower Soup
**Sydney**		
Main	Barley Risotto with Spinach, Artichokes and Sundried Tomatoes	Western Australian Sunkissed Tomato Barley Risotto
Soup	Tomato and Capsicum Soup	Queensland Roasted Tomato and Charred Capsicum Soup
Salad	Iceberg Lettuce Salad	English Garden Salad
**São Paulo**		
Main	Bean and Veggies Stew	French Slow-Cooked Mushrooms and Beans Cassoulet
Side	Taro with Herbs	Herb Roasted Tropical Potatoes
Soup	Collard Greens Vegetable Soup	Sweet Velvety Soup with Collard Greens

### Study sites, cafeterias and population

The study had a quasi-experimental design. It was conducted in four workplace offices in different parts of the world (Sydney, Chicago, Singapore & São Paulo) with onsite self-service, buffet-style cafeterias. All food in these cafeterias (i.e., all meals, beverages, and snacks) was provided complimentary to employees. The criteria for the selected locations were 1) to represent different continents and cultures; 2) to have at least two cafeterias on campus so one could be used for the intervention and the other for the control measurements; 3) to display the menus in English to ensure the consistency of the naming workshop; and 4) to serve a variety of plant-rich and meat dishes so that diners had adequate choices. The seated headcount at the test locations was ~ 850–2500 employees. About 50–60% were females, the mean employee age range was 28–40 and the job functions included sales, engineering, business and support services (mostly finance, operations and human resources).

### Timeframe and test dishes

The testing was conducted during lunch time at all sites from September to December 2019 (July to December in Chicago). Three plant-rich (vegan or vegetarian) menu items including main dishes, side dishes, composed salads and/or soups were tested at each site (Table 1). The dishes that were selected were expected to have medium popularity (based on past menu items) and had the potential for good appealing names to be created. The effect size that determined our sample size i.e., the number of total plant-rich dish showings to be tested based on four dish repetitions, was calculated based on a previous hypothetical online study conducted solely for this purpose (data not shown). Meat-based versions of the plant-rich test dishes were also tested to assess potential substitution effects (Table 2). Intervention cafeterias offered the test dishes once over a four-week menu cycle. During each menu cycle the menu choices changed daily and each cycle repeated four times a year (six times in Chicago only). This determined the total number of repetitions for each dish to be four (six in Chicago), meaning the plant-rich dishes were shown with their basic names two times (three in Chicago) and with their appealing names another two (three in Chicago). No changes to the dish preparation were made across dish repetitions and the only thing that changed during the study period was the dish title. The test dishes were new to the participants when first introduced.

**Table 2:** Meat-based test dishes along with their plant-rich dish pairs

Dish Type	Meat Dish	Paired Plant-rich Dish
**Chicago**		
Main	Roasted Lamb and Onions	Eggplant and Chickpea Stew
Main	Beef Stew	Seitan Stew
Soup	Beef and Bean Chili	Tomato Soup
**Singapore**		
Main	Beef Stew	Steamed Lentil & Couscous with Cauliflower
Main	Tofu & Chicken with Creamy Mustard Sauce	Steamed Lentil & Couscous with Cauliflower
Soup	Bacon, Leek and Potato Soup	Cauliflower Soup
**Sydney**		
Main	Beef Skewers with Herb Sauce	Barley Risotto with Spinach, Artichokes and Sundried Tomatoes
Main	Lamb with Herbs	Barley Risotto with Spinach, Artichokes and Sundried Tomatoes
**São Paulo**		
Main	Roasted Beef with Vegetables	Bean and Veggies Stew
Soup	Beef Soup with Cassava and Sausage	Collard Greens Vegetable Soup

Soups were tested both as test and control dishes to control for any seasonality or weather effect on dish choice. Control soups were offered at a different cafeteria in the same building to the intervention cafeteria. The cafeterias were on different floors in the building (except in São Paulo). All employees could access both cafeterias without restriction. The control soups were identical with the test soups at each location (except in São Paulo), appeared only with their basic name and were offered on the same day with the test soups. In São Paulo two different plant-rich soups were offered as the control cafeteria was on the same floor as the intervention cafeteria (intervention soup: collard greens vegetable soup; control soup: cauliflower cream with coconut).

### Intervention

During the intervention, each menu item appeared four times in total (six in Chicago as we had the opportunity at this site to test for a longer period of time) across repeated menu cycles as described above. The only exception was the barley risotto with spinach, artichokes and sundried tomatoes in Sydney that was presented twice within each menu cycle instead of once and eight times in total. Plant-rich main dishes and soups, along with their paired meat-based dish (and control dish for soups), were tested on the same day. All dishes were presented on the menu with their basic name the very first time (baseline) and then the plant-rich test dishes were presented alternating between basic and appealing names in a random order. For example, at one location the naming of the test dishes could have been basic/appealing/basic/appealing while at another site it may have been basic/basic/appealing/appealing. This was done to remove any naming order or time/date effects from the effect of the name. In all cases, all dishes were presented with their basic names as many times as with their appealing names. The meat-based dishes and control soups were presented with their basic names for all dish repetitions on the menu. Food service operators were specifically asked to not talk the test dishes up or down to the cafeteria diners.

### Measurements

For each test dish, the overall amount of food produced as well as any leftovers in the kitchen or the counters was weighed at the end of the service. From their difference the amount of food taken by the diners was calculated. In addition, the order at which the test dish appeared as well as the number of other items at the buffet line was recorded. The number of clean plates each cafeteria had available for diners to use during service and the number of clean plates left over at the end of the service were counted. From their difference the total number of plates taken by the diners was calculated. This was a proxy measure for how many diners served themselves from the cafeteria. The amount of food taken per plate was calculated by dividing the amount taken for each test dish by the number of total plates taken in the cafeteria. This measure was used in the analysis to account for differences in attendance at the cafeteria at each test day.

### Statistical analysis

To test the main study hypothesis, i.e., that selection of plant-rich dishes (food amount taken per plate) at the intervention cafeteria would increase when appealing names were used compared to basic names, we opted a priori to pool data across all four sites instead of analysing each location individually. This approach allowed us to maximize statistical power given that the study design was constant across all four sites. To account for the clustered nature of these data, we employed a linear mixed effects model using the lme4 package in R, with random-effects terms for dish and location. In order to reduce the noise of the data, the results were analyzed as percentage change from baseline i.e., the aggregated data for all basic (or appealing) names for each dish were analyzed as percentage change from their very first presentation that had a basic name. The results remained consistent when analyzing the amount of food per plate as grams/plate (data for grams/plate could be seen in Table 3). We further repeated the analysis using different covariates: month of year, order of presentation, number of other items in the buffet line, and day of the week. None of these covariates reduced the effect to non-significant. All tests were two-sided.

A number of exploratory analyses were also conducted using mixed effects models with random effects terms for dish and/or location. First, we investigated our secondary hypothesis whether the selection (food amount taken per plate) of meat-based dishes was decreased when paired with plant-rich dishes with appealing names (with random effects terms for dish and location). Second, using a random effects term for dish, we tested for each site separately whether there were differences in the selection of plant-rich dishes (food amount taken per plate) between appealing and basic names. Third, we tested whether the effect was more prominent for specific dish types and, most importantly, for soups, which were the only options to be offered in opaque containers with closed lids, thus removing the influence of any visual and olfactory food cues on dish choice (with random effects term for location). Data was not analyzed separately for salads as there were only four showings of them. In addition, we tested whether the effect was stronger or weaker depending on the popularity of the dish at baseline. Finally, we tested whether food consumption at the control cafeterias significantly changed compared to baseline via a mixed effects model with a random effects term for dish (as there was only one control dish per site, we did not use a random effects term for location). All tests were two-sided.

## Results

**Fig. 2:**
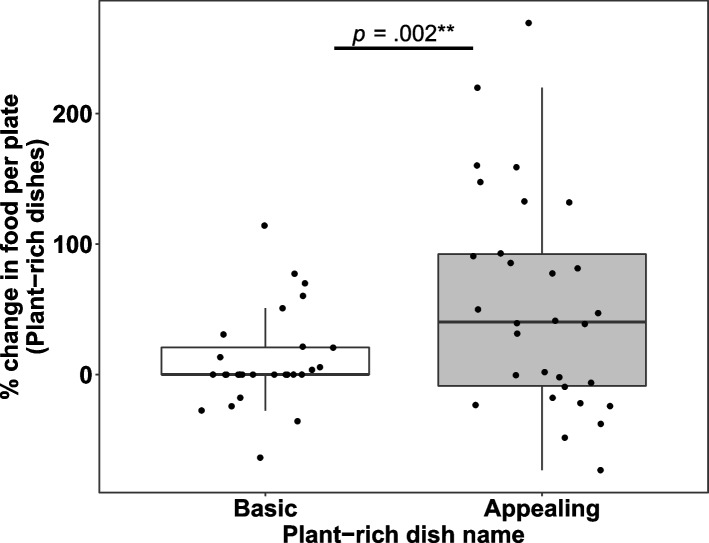
Percentage change of the food amount taken per plate relative to baseline for the basic (*n* = 28) and appealing (*n* = 30) names of plant-rich dishes. The food amount taken was estimated by dividing the total amount of food taken for each dish by the total plate count from that lunch period. The food amount taken was then normalized to the first presentation of that dish (which always had a basic name)

Overall, appealing dish names significantly increased the amount of food taken per plate by 43.9% relative to baseline where basic dish names were presented (54.5% vs. 10.6% increase for appealing vs. basic names, respectively, *b* = 39.32, *SE* = 12.20, *t*(45.69) = 3.22, *p* = .002; Fig. 2). In real terms, this corresponded to a 7% increase in food amount taken (27.6 g per plate vs. 25.8 g per plate for appealing names vs. basic names, respectively, *b* = 2.88, *SE* = 1.65, *t*(44.91) = 1.75, *p* = .087; for all results in actual grams see Table 3). The results remained significant when controlling for the number of other items served alongside the intervention dish, the relative order of the intervention dish (i.e., first, second, last, etc.), the day of the week and the month of the year.

**Table 3:** Amount of food taken per dish and percentage change for appealing and basic names

Dish Type	Basic Name	Appealing Name	Amount of food taken at baseline *g/plate*	Average amount of food taken for basic names (including baseline) *g/plate (% change from baseline)*	Average amount of food taken for appealing names *g/plate (% change from baseline)*	Difference between amount of food taken for appealing versus basic names including baseline *g/plate (% change of appealing names from basic names)*
**Chicago**						
Main	Eggplant and Chickpea Stew	Nonna’s Garden Ragout	4.26	6.87 (+ 61.38%)	12.21 (+ 187.03%)	5.34 (+ 77.86%)
Main	Seitan Stew	Wine Simmered French Vegetable Medley	7.86	9.98 (+ 26.99%)	10.34 (+ 31.46%)	0.36 (+ 3.52%)
Soup	Tomato Soup	Provencal Slow-Roasted Herbal Tomato Soup	4.87	5.37 (+ 10.33%)	12.89 (+ 164.89%)	7.52 (+ 140.09%)
**Singapore**						
Main	Steamed Lentil & Couscous with Cauliflower	Soft Baked Cauliflower Tossed with Moroccan Grains	63.84	58.18 (−8.86%)	48.69 (−23.73%)	−9.49 (−16.32%)
Side	Steamed Mixed Vegetables	Tricolore Summer Vegetables	57.89	50.86 (−12.14%)	50.94 (−12.01%)	0.08 (+ 0.15%)
Soup	Cauliflower Soup	French Smoked Cauliflower Soup	37.53	38.59 (+ 2.83%)	41.67 (+ 11.03%)	3.08 (+ 7.98%)
**Sydney**						
Main	Barley Risotto with Spinach, Artichokes and Sundried Tomatoes	Western Australian Sunkissed Tomato Barley Risotto	28.93	36.48 (+ 26.11%)	44.13 (+ 52.56%)	7.65 (+ 20.97%)
Soup	Tomato and Capsicum Soup	Queensland Roasted Tomato and Charred Capsicum Soup	50.16	43.26 (−13.76%)	44.15 (− 11.99%)	0.89 (+ 2.05%)
Salad	Iceberg Lettuce Salad	English Garden Salad	11.27	12.46 (+ 10.72%)	18.45 (+ 63.73%)	5.98 (+ 47.88%)
**São Paulo**						
Main	Bean and Veggies Stew	French Slow-Cooked Mushrooms and Beans Cassoulet	18.30	22.96 (+ 25.44%)	30.44 (+ 66.31%)	7.49 (+ 32.59%)
Side	Taro with Herbs	Herb Roasted Tropical Potatoes	24.78	20.36 (−17.87%)	14.11 (−43.07%)	−6.24 (−30.69%)
Soup	Collard Greens Vegetable Soup	Sweet Velvety Soup with Collard Greens	16.23	11.07 (−31.80%)	16.89 (+ 4.10%)	5.82 (+ 52.64%)

**Fig. 3:**
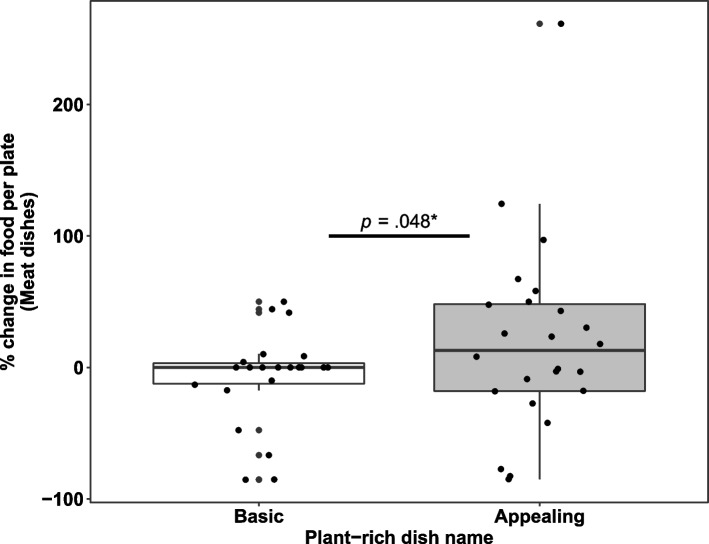
Percentage change of the food amount taken per plate relative to baseline for meat dishes when the corresponding plant-rich dish names were either basic (*n* = 22) or appealing (*n* = 24). The food amount taken was estimated by dividing the total amount of food taken for each dish by the total plate count from that lunch period. The food amount taken was then normalized to the first presentation of that dish. Meat dishes were always presented with basic names

No evidence was found from secondary analysis to support a decrease in meat selection when plant-rich dishes were presented with appealing names compared to basic names. On the contrary, meat selection expressed as percentage change from baseline was slightly increased when plant-rich dishes had appealing names (20.3% increase vs. -12.8% decrease for appealing vs. basic plant-rich dish names, respectively, *b* = 25.30, *SE* = 12.32, *t*(35.43) = 2.05, *p* = .048). However, this difference disappeared when analyzing the grams taken per plate (42.1 g of meat/plate vs. 37.8 g of meat/plate for appealing vs. basic plant-rich dish names, respectively, *b* = 3.30, *SE* = 3.02, *t*(34.96) = 1.09, *p* = .283). In other words, this result was not consistent between the different measures of consumption (when analyzing percentage change the effect was barely significant, but when analyzing the grams taken the effect was not significant) and shall be interpreted with caution. The results did not change when covariates were included in the analysis (Fig. 3).

**Fig. 4:**
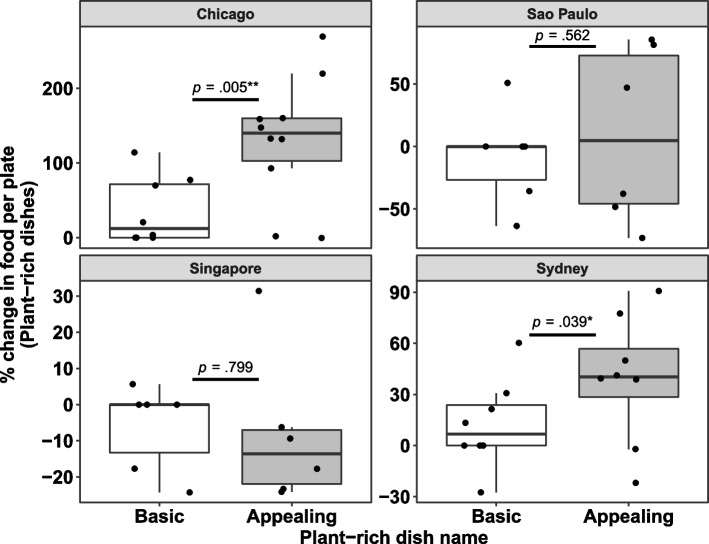
Percentage change of the food amount taken per plate relative to baseline for the basic and appealing names of plant-rich dishes by study location. Sample sizes: Chicago *n* = 18 (8 basic names, 10 appealing names), São Paulo *n* = 12 (6 basic names, 6 appealing names), Singapore n = 12 (6 basic names, 6 appealing names), Sydney *n* = 16 (8 basic names, 8 appealing names). The food amount taken was estimated by dividing the total amount of food taken for each dish by the total plate count from that lunch period. The food amount taken was then normalized to the first presentation of that dish (which always had a basic name)

Secondary analysis revealed that the effect was site specific (Fig. 4). Specifically, the effect was statistically significant in the two sites where English was the native language for the majority of employees i.e., Chicago and Sydney. Appealing names increased the amount of food taken per plate relative to baseline by 74.3% in Chicago (131.5% vs. 57.2% increase for appealing vs. basic names, respectively, *b* = 93.34, *SE* = 28.52, *t*(14.18) = 2.27, *p* = .005) and by 19.5% in Sydney (39.2% vs. 19.7% increase for appealing vs. basic names, respectively, *b* = 26.92, *SE* = 11.63, *t*(11.96) = 2.31, *p* = .039). The effect was not significant in São Paulo (9.1% increase vs. -16.2% decrease for appealing vs. basic names, respectively, *b* = 17.19, *SE* = 28.43, *t*(8) = .61, *p* = .562) or in Singapore (− 8.2% decrease vs. -12.1% decrease for appealing vs. basic names, respectively, *b* = − 2.18, *SE* = 8.27, *t*(8) = −.26, *p* = .799). The results did not change when covariates were included in the analyses.

**Fig. 5:**
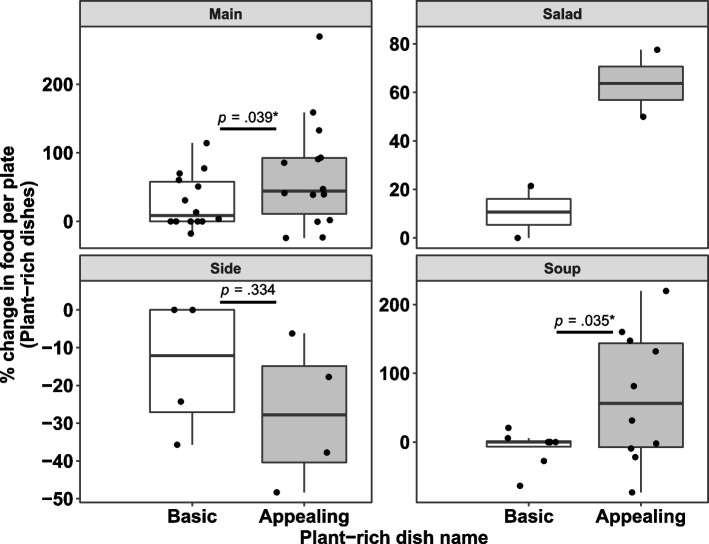
Percentage change of the food amount taken per plate relative to baseline for the basic and appealing names of plant-rich dishes by dish type. Sample sizes: Main *n* = 28 (*n* = 14 for basic names, n = 14 for appealing names), Salad *n* = 4 (n = 2 for basic names, n = 2 for appealing names), Side *n* = 8 (n = 4 for basic names, n = 4 for appealing names), Soup *n* = 18 (n = 8 for basic names, *n* = 10 for appealing names). The food amount taken was estimated by dividing the total amount of food taken for each dish by the total plate count from that lunch period. The food amount taken was then normalized to the first presentation of that dish (which always had a basic name). Statistical tests were omitted for salads due to low sample size

A significant effect of appealing names was found for main dishes (b = 39.14, SE = 17.86, *t*(21.97) = 2.19, *p* = .039) and soups (b = 61.04, SE = 26.02, *t*(13.30) = 2.35, *p* = .035), resulting in significantly greater dish selection compared to the basic names (Fig. 5). Only four observations were available for salad dishes, therefore we did not run statistical tests on these dishes alone (although we noted the pattern was directionally consistent with our hypothesized effect). No significant effect was found between the appealing and basic names for side dishes (b = − 12.54, SE = 11.73, *t*(5) = − 1.07, *p* = .334; Fig. 5). It is, however, important to note that side dishes were only tested in São Paulo and Singapore, the two sites that showed no overall effect of the intervention. It should also be noted that this analysis did not have sufficient statistical power, suggesting the results shall be interpreted with caution.

In addition, we found that the effect of appealing names compared to basic names was stronger for dishes that were less popular at baseline i.e., dishes at -1SD on volume taken at baseline (*b* = − 1873.25, *SE* = 575.45, *t*(44.75) = − 3.26, *p* = .002.). Finally, there was no significant change in selection of control dishes over time (*b* = − 63.16, *SE* = 62.33, *t*(20.27) = − 1.01, *p* = .323).

## Discussion

In this real-world study, we tested a naming approach to promote plant-rich dishes using appealing descriptions. Overall, our results showed that appealing dish names increased the amount of food of plant-rich dishes taken by diners at two of the four global workplace sites. In order to understand the applicability of this strategy to geographies and cultures beyond the US and the UK, we examined the effect of appealing dish names in different global locations including Asia, Pacific and Latin America. The effect appeared significant only at sites where English was the native language. In addition, when we tested for a substitution effect between plant-rich and meat dishes we did not find any; meaning people were not selecting a plant-rich dish in the place of a meat dish. While the intervention effect was modest, scaling the strategy across food service companies could have a tremendous impact on shifting diets towards more plant-rich choices as a 7% increase could correspond to many thousands of meals if applied broadly.

The findings agree with previously published research showing that appealing names can increase the selection or sale of plant-rich dishes. Specifically, naming the vegetable dishes served at US university cafeterias with indulgent or taste-focused descriptors led to significantly more people choosing the vegetable dish compared to basic or health-related descriptors [15, 16]. In another experiment conducted in the UK’s second largest grocery store chain onsite cafeterias, descriptors emphasizing the dish taste, origin and those making the dish sound more appealing significantly increased the sales of plant-rich dishes compared to basic names [18]. On the other hand, naming dishes as vegetarian has been shown to lead to decreased selection [19]. Although direct comparisons of the magnitude of the effects are difficult to make between studies due to heterogeneity in design and measurement, taken together these findings indicate that using appealing dish names at the point of decision show some promise as a means to promote more plant-rich food choices in food service.

Potential mechanisms that could explain why appealing names influence selection can be identified from existing research on advertising and mental simulation. For instance, appealing menu names may alter taste perceptions [20], trigger tempting mental imagery [21], trigger positive mental simulations of eating and evoke positive expectations (e.g., due to vivid recall and imagining of eating the food, including the taste and texture of the food, eating situations and hedonic enjoyment [22, 23]).

Although appealing descriptors can attract consumers to the dish, repeat selection of plant-rich dishes depends on whether or not dish expectations created by the appealing names are met after tasting. Moderation analysis has shown that tastier vegetable recipes drive stronger effects [15]. However, the type of expectation generated by appealing names was not examined in the current study and warrants further investigation. Nevertheless, making plant-rich dishes delicious in combination with appealing dish names holds promise as the recipe for success. In addition, renewing appealing names systematically could also keep diners interested in them as a diminishing effect might be observed with repeated name showings. Again, the validity of this hypothesis and identifying the optimal frequency for changing the names require further investigation.

A secondary finding from the present study was that the effect of appealing language was observed only at sites where English was the native language. This finding may reflect the close relationship between language and culture [24]. Past work has found that linguistic and cultural differences are important factors that affect the way people respond in multi-country interventions [25]. For example, a major driver for food choices in western countries is taste. Consumer research shows that taste is the top driver of purchase decisions (88%), a finding that has not changed over the past decade [26]. However, this is not the case in all cultures. In China for example, far greater importance is attached to foods that evoke a sense of honor and prestige [27]. In addition, a study [28] comparing motives for food choice in different Asian and Pacific countries showed much heterogeneity in food choice drivers. Particularly, the most important driver in Taiwanese and (ethnically Chinese) Malaysian consumers were health, natural content, weight control and convenience, in Japanese consumers was price and in New Zealand consumers was sensory appeal. In addition, the variable of ‘familiarity’, which was a key focus in our naming workshop, was rated as least important by all countries. This fact demonstrates that consumers from different cultures have different drivers when it comes to food choice [28]. In other words, because our naming approach for developing appealing names was tailored to western-centric cultures, its effectiveness did not seem to generalize well to other cultures. This suggests that when global initiatives and strategies are being designed, guidelines and tool attributes need to be adapted to different cultures before scaling across different geographies.

Another important aspect that may have muted the effectiveness of this approach is that people do not always read the names of dishes served from a buffet. Instead, they often rely on what they see and smell when choosing what to put on their plates. Visual cues have a strong effect on guiding food choices [29]. In the current study, the best way to increase internal validity and examine the isolated effect of appealing dish names was via soups. This was because visual and olfactory cues were removed as soups were offered in opaque pots with closed lids and people had to read the labels to understand the soup on offer. In the present study, in three out of the four locations (i.e., Chicago, Singapore and São Paulo), the increase in soup selection with the appealing names was larger than the increase in other types of dishes where consumers could see the dishes they were selecting. However, given that a significant effect was also observed for other types of dishes, no robust conclusions can be drawn. Nevertheless, this indicative evidence supports our hypothesis about appealing dish names attracting consumers to plant-rich dishes in the absence of other sensory cues.

No evidence was found that diners substitute meat dishes with plant-rich dishes even when the latter were presented with appealing names. Although appealing names increased the selection of plant-rich dishes, this increase was additive and not a replacement to the selection of meat dishes. This may increase the overall energy consumption from the meal and this is something that warrants further investigation.

### Strength and limitations

Our study examined a systematic naming approach to improve the appeal of plant-rich dishes. To date, most of the existing research on this topic has been conducted in the US and the UK. For the first time, we examined how this approach performed across other geographies and cultures. In addition, most of the research into the role of appealing language on food choice has been conducted either online or in academic cafeterias or restaurant settings. This study examined the effect of appealing dish titles in a workplace dining environment. Workplaces are important settings to consider as people often spend a significant amount of time at work and regularly consume a meal there. Additional strengths of the study include the elimination of the influence of dish price, which is a major determinant of food selection as food was complimentary at the point of service in this study. Notably, it is important for future research to examine the interaction between appealing names and pricing as people might select in a different way when they have to pay. The repeated menu cycles also provided the opportunity to test the same dish across different time points, thus improving reliability of the findings, as well as studying dishes from different cuisines and different types i.e., mains, sides, salads, soups. Limitations of the study include the inability to implement a fully controlled experimental design given the applied nature of this research, although control dishes were used in every location. The quasi-experimental design provides low internal validity but, on the other hand, maximizes external validity. In addition, a test to verify whether the appealing names were considered appealing by the audience would be helpful to provide further confirmation for the efficacy of the workshop. Lastly, the sample size was not big enough for the secondary analysis performed, increasing the likelihood of type 2 error in our interpretations i.e., finding a false null result and no firm conclusions can be drawn. Conversely, power for the main analysis was adequate to detect significant results.

## Conclusions

In conclusion, this study indicates that a systematic approach to generating appealing names to promote plant-rich dishes in a real-life dining environment can be effective in influencing diners’ food choices. It also shows that the effectiveness of this strategy is influenced by the cultural and linguistic context and that adaptations must be made before applying the approach in different locations. Appealing dish names are a relatively easy, cost-effective, scalable strategy that food service providers could adopt to shift food choices. Applying this to workplace environments, as well as more broadly, could affect millions of meals on a daily basis and induce large scale shifts to more plant-rich dishes.

## Data Availability

The datasets used and/or analysed during the current study are available from the corresponding author on reasonable request.
